# Considerations on the Nature and Treatment of Some Hereditary or Family Diseases

**Published:** 1809-03

**Authors:** M. Portal

**Affiliations:** Professor of Medicine in the College of France, and of human Anatomy in the Museum of Natural History, Member of the National Institute, &c. &c.


### *Considerations on the Nature and Treatment of some hereditary or Family Diseases; by* M. Portal, *Professor of Medicine in the College of France, and of human Anatomy in the Museum of Natural History, Member of the National Institute, &c. &c.*

Maxima ortus nostri vis est, nec parum felices bene nati.

*Fernel de Morbor. Causis Pathol. Lib. I. cap. xi.*

Read before the French Institute, Jan. 25, 1808.

It cannot be disputed that there are diseases which are transmitted from parent to child; while the latter inherits the general exterior resemblance of his father, or even his shape, characteristic traits,* looks,† or voice,‡ he also inherits his father's health, strength, and sometimes his diseases. Thus Fernel, the celebrated physician of Paris, observes, *Maxima ortus nostri vis est, nec parum felices bene nati.* It cannot be denied that there are also families, the individuals of which attain a greater age than others; and this circumstance has occasioned a remark, that there are short as well as long lived families. ||

We may safely add, that if children have a physical resemblance to the parents, they resemble them in their moral character also. "We find," says Montaigne, "that not only are the marks of the body transmitted from father to son, but also a resemblance of temper, complexion, and inclinations of the mind."§ This is well ascertained by the result of examples which frequently fall under our observation; and is not one of these resemblances, physical or moral, a natural consequence

---

* These include the colour of the skin, hair, and eyebrows, with the form of the body, attitudes, gestures, and gait.

† At Montmorency there exists a kind of strabismus. The family of Messrs. Nanteuil, director of the posts, is remarkable for enormous black eyebrows, &c. This is one of the many examples which we witness every day.

‡ All the Messrs. Garat have fine voices, and so closely resembling each other, that when they sing or talk, they can scarcely be distinguished from each other. It has been thought, that in this family the peculiarity of voice was transmitted by their mother, who was remarkable for a very fine voice, and which strongly resembled that of her children. There are some families, the individuals of which are remarkable for good or bad teeth.

|| Haller has cited some examples of longevity, or of shortness of life in several families; every person is acquainted with similar instances.

§ Mores ingenerantur a stirpe generis. Baillou de Calculo.

consequence of the other.* Would not the moral resemblance be more striking and more frequent, if education did not produce a difference ?†

We may venture to assert, that nature originally formed mankind in the most perfect manner possible, as well as all other beings, both as to the structure of the different parts, their configuration, size, and relative situations. Thus man, in the state of nature, would enjoy the best health, the greatest strength, and the finest and most regular shape: finally, the moral faculties would exist in all their energy, if some extraneous cause did not intervene to weaken them; can we refuse to admit this as an incontrovertible axiom ?

But there are many causes which derange this admirable harmony; may not the parents previous to their marriage have contracted diseases, which have occasioned in their organs real affections, rendering them totally different to what they were in a state of nature ? Thus, at the moment of procreation, they receive the different characteristics of their parents, which they have unfortunately acquired, and which they may transmit to their progeny. Has not the mother during pregnancy an influence over the infant in her womb, either by assimilating it to herself in some measure by the nourishment she gives it, or by causing it to feel a part of the evils she herself experiences, and communicating some impressions resulting from these causes ?‡

The infant on coming into the world, may be very different from what it would have been had these causes not existed,

---

* ——— Gigni pariter cum corpore et una,
Crescere sentimus, pariter senescere mentem.
LUCRETIUS.

† Every person can adduce instances of families, the children of which are ingenious, and disposed to profit by instruction; whilst other children are dull, and incapable of making any progress in education. I am acquainted with families, the children of which have had the best masters, and yet they have derived no advantage from their instruction for the want of genius. The number of this class of human beings is greater than is generally supposed.

‡ Are not spots upon the skin more or less extensive and variously coloured, fungous excrescences of all kinds, and the various marks upon the skin, which resemble fruit, animals, &c. the effects of painful breedings, and other disagreeable circumstances attending the mother? But if alterations may be produced on the skin, may not the internal parts, which are less liable to inspection, be subject to similar phenomena ? This is more than probable; and does it not follow, that in their moral and physical dispositions, children resemble the mother more than the father ?

existed, which are as it were extraneous to it, and which make it differ from its parents as to their primitive state of health, and make it resemble them in their diseases; and as the number and inveteracy of diseases increase as men advance in life, however strong or healthy they may have been originally, the children of old men are more subject to hereditary diseases, and their constitutions more feeble.* The nursing of the child by its own mother, or by a strange nurse, may also produce other differences more or less remarkable, with respect to its physical or moral constitution, but which will produce a resemblance to its nurse. Thus the ancients, who regarded the nurse as a second mother, comprehended among hereditary diseases the *morbi congeniti, cognati, seu connutriti* of Hippocrates, the *morbi parentales* of Pliny, the *hæreditarii* of Fernel, and those which infants contract from their nurses; and in fact, these are too frequently conspicuous.

Hippocrates, Galen, Fernel, Ingrassias, Baillou, Lazare Riviere, Mead, Boerhaave, Morgagni, Stahl, Senac, Lieutaud, Haller, Zeller, Van Swieten,† and other great physicians,‡ whom it would be futile to name after them, have admitted of hereditary or family diseases, and have included in this class, scrophula, rachitis, mania, epilepsy, convulsions, apoplexy, paralysis, diseases of dentition, pulmonary consumption, asthma, dropsy, gout, and stone in the bladder; and can there be a single practitioner, more especially in a great city, where the examples of these diseases are more numerous, who is not convinced from personal observation, that the children of parents who have been subject to these diseases, have generally inherited them? We say generally, because there are numerous

---

* Senes et valetudinarii, imbecilles, filios vitiosa constitutione gignunt, quâ tandem in morbos similes hereditarios idcirco nuncupatos, incurrunt, ut parentibus liberi succedant, non minus morborum, quam possessionum hæredes. *Fernel,* De morborum causis, lib. i. cap. 11.

† Boerhaave, *Aphor. de curandis morbis,* 1075; Van Swieten, *ibid. Morbos ex parentibus propagari in progeniem, innumeris observationibus confirmatur.* Aphor. 1198, t. IV. p. 16. These authors were of opinion, with some of their predecessors, that diseases were transmitted to grand-children, without breaking out in the intermediate generation. *Silente sæpe morbo in genitore dum ex avo derivatur in nepotem. Aphor,* 1075; and this opinion of Boerhaave is confirmed both by exterior resemblances and by family diseases.

‡ M. Forestier has published within these few years, an excellent dissertation, *De morbis aut noxis puerorum in vitiatis, depravatisve parentibus.*

rous exceptions on this head, even when the legitimacy of birth cannot be questioned.

To these hereditary diseases may we not add cancer and cataract,* with deafness and dumbness from the birth? Morgagni saw three sisters who were dumb from their infancy. Other authors have mentioned similar instances; and many such have come under our own observation. Those acquainted with herniary complaints do not hesitate to affirm that hernia is more frequent in some families than in others; so far, therefore, from limiting the number of hereditary diseases, and still farther from denying their existence entirely, as some authors have done, we think their numbers are very considerable.† Without, however, wishing to go to the extent that Hippocrates has done, who was of opinion, that all diseases were hereditary, *aliqua quidem ex parte*;‡ and that all children inherited, more or less, the temperament of their fathers. §

The opinion of Hippocrates has been followed by all except Sennert, Ethmuller, and Maurice Hoffman, who do not admit of any acute diseases being hereditary. As to the transmission of chronic diseases from father to son, they have regarded it not only as possible, but as very common; || and this doctrine was so generally adopted in 1748, that the Academy of Dijon proposed a prize question, with a view to determine *how the transmission took place*. M. Louis, who subsequently became so celebrated in the Annals of French Surgery, instead of answering the question proposed, published a well written Dissertation, in order to prove that there were no hereditary diseases; but his arguments on this subject are more ingenious than well founded.

---

* Woolhouse de la Cataracte, p. 24. See a remarkable instance in the Journal de Paris, article *Evreux*, 13 Dec. 1807. I have seen three children out of four, in one family, who were blind from their birth by an amaurosis or gutta serena.

† Haller has admitted of a very great number. *Physiol. Elementa de Semine.* Lib. xxix. Sect. ii. Art. vii.

‡ Prædict. Lib. ii.

§ Ex pituitoso, pituitosus, ex biloso biliosus gignitur, ut ex tabido, tabidus et ex lienoso lienosus; quid prohibit ut cujus pater et mater hoc morbo correpti fuerunt, etiam posteriorum ac nepotum aliquis eo corripiatur; semen enim genitale ab omnibus corporis partibus procedit, a sanis sanum, a morbosis morbosum. *Hipp. de Morbo Sacro.*

|| Stahl admits of a certain predisposition to various diseases in different families.

The

The difficulty, or rather the impossibility of a satisfactory explanation of the communication of diseases from parents to children, has more than once given occasion to medical authors, to deny the existence of hereditary taints; as if it were always necessary, before admitting an effect, to know its cause; and yet, by a strange contradiction, the same writers do not hesitate to recognize the external resemblance between children and their parents, which they cannot account for. *Rerum eventa magis arbitror, quam causas,* says Cicero, *quæri oportere; et hoc sum contentus quod etiam si quomodo quidquid fiat ignorem, quod fiat intelligo.*

Let us study the phenomena of Nature, even when she conceals from us the means she employs for producing them; to be acquainted with them is always curious, and it is useful if it facilitates the progress of the healing art.

The Royal Society of Medicine gave out, as Questions for the Prize Dissertations for 1787. 1st. Do hereditary diseases exist, and what are they? 2d. Is it in the power of medicine to hinder their developement, or to cure them when they have broken out?

Some of the memoirs presented on this occasion have been printed, but what their authors have said, does not appear to us to have exhausted the subject. The present remarks being the result of our clinical and anatomical observations, prove that there are family or hereditary diseases, and also seem to lead us to the knowledge of the nature and treatment of several of these diseases.

Hereditary disease consists not only in mal-conformations, more or less extensive of the external parts, but frequently also of internal deformities, and which dissection alone can demonstrate; it is from these internal mal-conformations, and also from peculiarities in structure, that the alterations of the functions, or the various symptomatic hereditary diseases, proceed. We shall endeavour to prove this in the following pages.

After mentioning such mal-conformations as are external, we shall proceed to speak of those which we have discovered in the internal parts.

It cannot be denied, that there are families, the individuals of which have larger heads than usual. There are some also, but not so common, who have small heads and large bodies: at other times, and in the same family, we meet with craniums, long, narrow, broad, short, or high in proportion; this, however, is of no consequence relative relative to the moral and physical constitution, if the capacity of the cranium be the same, which is generally the case, as Hippocrates and other accurate observers have remarked.*

To return to the differences observed in families:— The children of some parents have the nasal bones and cartilages more elevated or flattened;† shorter or longer; and more or less covered with a fatty substance. Hence it follows, that the individuals of certain families have noses of a form and size which distinguish them from others; thus, the family to which Charles Boromæus belonged, was remarkable for large aquiline noses, which are to this day observable in his descendants.‡ The Bourbons

* Nothing can be more fallacious than to judge of the capacity of the cranium from the bulk and form of the head, the bones being sometimes very thick, and sometimes very thin, and covered with muscles, so as to give more or less bulk to some parts of the head. Frequently when the cranium is convex on one side, it is proportionably flat on the other: this led Riolamus to blame those ancients, who appreciated according to the size or figure of the cranium, the strength, weakness, rectitude, or depravity of the mind.

It cannot, however, be denied, that there are some malconformations of the cranium, which injure the functions of the brain, and a defect or irregularity in the developement of the bones of the cranium in infancy may be the original cause. Hulnaud has remarked, that when ossification goes on too rapidly in children, the sutures disappear, and the bones unite into one solid piece: hence the cavity of the cranium ceases to increase, at least so far as to prevent the brain from assuming its free and complete developement; hence there must result a compression upon that organ, and consequently a derangement of the physical and moral functions. Of this, in my opinion, there cannot be a doubt; but is not the scrophulous tendency in some families, a frequent cause of all these disorders, in the developement of the parts, and of the changes in their structure? In my *Anatomie Medicale* I have cited some facts which prove this completely.

† Infants have also very flat noses, from the frontal sinuses not being as yet developed; but when the anterior laminæ of these sinuses come forward as a consequence of their growth, which is greatly favoured by the process of respiration, the root of the nose rises more or less, and on this subject there are great varieties: in some families we see the root of the nose almost upon a level with the *glabella*, or that part of the forehead situated between the eye-brows, and in other individuals it is sunk below that level: these differences give rise to peculiarities in point of physiognomy, which must have been observed by every person.

‡ Dr. Gregory, one of our old pupils, and now the distinguished Professor of the Theory and Practice of Medicine at Edinburgh, related to his numerous students, in order to convince them of the resemblance between children and parents, both with respect to external and internal structure, that having been once called to a distant part of Scotland, to visit a rich nobleman, he discovered in the configuration of his nose an exact resemblance to that of the Grand Chancellor of Scotland in the reign of bons have all large noses, and the individuals belonging to the Austrian branch have thick lips. I have known families, in which the ears were very large, and in others in which they were small, and almost without any lobulæ.

There are individuals also, the bones of whose faces are more or less convex, the lower part of the chin hollow or elevated; the face more or less oval, or irregularly triangular or square; more prominent or flat, and sometimes as if truncated at the lower extremity from a defect in the development of the lower jaw.

In certain families the individuals have capacious chests, and in others this cavity is narrow and contracted; some families have broad shoulders, others narrow, and this last defect coincides with that of a too narrow chest.

There are many families, the individuals of which are hunch-backed. I know one family at Paris, in which there are seven of this description; others have their limbs distorted, or too short, or too long, in proportion to the rest of their bodies.

There are persons also, with small or large hands, and with short or long feet. A man was once exhibited to us, at the Academy of Sciences, whose hands were of a monstrous size, and he assured us that his father's were equally large.

Some families, as mentioned by M. Morand, in a paper printed among the Memoirs of the Academy of Sciences (1769) were remarkable for having children with six fingers.*

External deformities in families have been noticed in all ages, and the ancients never doubted that they were hereditary. They were so convinced that children resembled their parents, that they used the terms *macrocephali à macrocephalis;* and the Romans, *capitones à capitonibus, pumiliones à pumilionibus.*

Independently of these differences with respect to the developement of the bones, we may remark in some families of Charles I. as represented in his portraits On taking a walk through the village after dinner, the Doctor recognized the same nose in several individuals among the country people; and the Nobleman's steward, who accompanied him, informed him, that all the persons he had seen were descended from the bastards of the Grand Chancellor. How many similar examples might we not adduce, if we were to pay attention to the subject?

* M. de Reaumur has cited the Kalleia family, as being remarkable for having six fingers on each hand, and six toes on each foot. Haller, Elementa Physiol. Vol. vii. Lib. 29.

lies real differences in the volume of the muscles of the trunk and of the limbs. I have seen instances where a father and his two sons had the left side of the body, with respect to the muscles, much larger than the right; they therefore used the left side and limbs more frequently than the right, and were of course left handed. In some persons the left side is stronger than the right, but this is very rare, for men of all countries are generally strongest in the right side.

I knew a family, the father and children of which possessed such a disposition in the muscles of the nose and of the lips, and such great mobility in the cartilage of the nose, that they could not speak without moving them. In the act of speaking, the point of the nose was constantly in motion.

I knew a Spanish nobleman who had one cheek larger than the other, the maxillary bone on one side and the integuments being larger than the natural size. He told me that his father and his uncles had a similar deformity, which was certified to me by several Spaniards then in Paris.

Some authors have described families of *triorchides*, or with three testicles, among which the Coglioni have been mentioned. On this subject, however, we should not forget, that we may sometimes mistake a preternatural tumour in the bursæ, or an epiplocele, for a testicle.

These external deformities ought to lead us to enquiries with respect to the interior of the human body.* May there not be natural or morbid relations between the internal and external parts? I have collected several examples of external resemblances in persons of the same family, who died of the same diseases; and I do not doubt, that if the inquiries now suggested are followed up, the results will every day prove more interesting. Future anatomical researches will show, that the viscera in the individuals of certain families were larger or smaller, and more or less different in their substance, so as to produce hereditary diseases.

Among several facts of this kind, which I have collected, I shall confine myself to two families; those of Vitel, Rue Saints Peres; and Villement, perfumer, Marche St. Martin,

* If we have been rather prolix on the subject of external resemblances, it is because we wish to impress upon anatomists that, upon examination, they will find the internal structure of the individuals of families equally striking with respect to resemblance.

Martin. In these families several individuals died of palpitation of the heart, after medical assistance had been administered in vain. I was present at the opening of the bodies of two of these patients, one in each family, and I saw that the left ventricle was very much dilated, altho' the parietes of this ventricle was enormously thick in both subjects. As other relations had also died under circumstances perfectly similar, we may reasonably suppose that if they had been opened, the same malconformation would have been found.

Palpitations of the heart, in consequence of aneurism, have been often noticed by authors, and particularly by Lancisi, who cites cases of this kind, which he witnessed in Italy, and which are still very frequent in that country. I have often been consulted myself by Italians for similar complaints,* The Gonzalvi family presents a striking example of this description, now under our observation.

Are there not also nervous and spasmodic affections in families, which derange the functions of the mind? or, do these functions remain unaffected during convulsions or inordinate exertions of the muscles!

How many families are there, in which mania, hysterics, and shakings of the limbs are hereditary? At Paris, we have seen the Marechal de Beauveau and four of his sisters, who were subject to very singular shakings of the head. It may perhaps be supposed, that this kind of convulsion was the effect of imitation from the parties frequently seeing each other, of which there are examples; but it could not be so in this case, for the parties were never long resident together in one place. It is also remarkable, that this shaking of the head attacked all of them at nearly the same age.

Morgagni has recorded the history of a family, some of the individuals of which died from excessive vomiting. In one of these persons, whose body was opened, the stomach

---

* I have no doubt that these hereditary palpitations are frequently occasioned by an increase in the thickness of the parietes of the ventricles, proceeding from a kind of steatomatous disposition: I do not think, however, that the parietes of the heart, although thicker, are stronger on that account, and that the aneurism is active, as has been of late asserted; for in that case the parietes of the heart, although thicker, on account of the disease, are weaker, and thereby more susceptible of being distended by the blood, the only agent in the dilatation of the heart, and of the vessels subject to aneurism: this is the reason why this aneurism is passive, as it is when the parietes of the heart are thin.

stomach was found shrivelled up, the pancreas hard, as if schirrous, and there were numerous concretions which united the pericardium to the heart.

There are families remarkable for having the epiploon enormously surcharged with fat, or with larger bellies than is requisite for their size in other respects;* this kind of malconformation is frequently followed by dropsy, and on opening the bodies of persons of this description, steatomatous concretions are generally found. I could adduce several examples in support of what I advance.

Do these hereditary diseases arise from various causes, or are they to be ascribed in most cases to one alone? This question seems worthy of some attention.

In the first place, it is certain, that several of these diseases are indicated by the external configuration of the bony parts, tending more or less to rachitis, which is of course propagated in families.

May not epileptic and maniacal persons have an external conformation; of the cranium for instance, which inclines more or less to rachitis?

Are not pulmonary phthises announced by the narrowness of the chest, a bad conformation of the sides, or the clavicles with a projection of the shoulders from behind, *(scapulæ alatæ)*? If these questions are answered in the affirmative, it follows that several hereditary diseases are more or less allied to rachitis.

This vicious conformation, however, does not exercise all its bad effects in a visible manner upon the osseous part of the trunk; it produces internal deformities. These are often discovered in the female pelvis, when the body appears in other respects well formed.

But rachitis, or the affection of the bones which alters their form, being an effect of the alteration in the lymph, well ascertained by the symptoms of the disease and by dissections, the alteration of these substances from the same cause may probably take place in other internal parts, without the bones being visibly affected. A thousand facts might be adduced in support of this opinion.

However different these diseases may appear, there is little doubt that they are the effects of one cause, differing only with respect to a few modifications, and with respect

---

* The Greeks called these persons *Physcones.* One of the Ptolemies was called the *Physco,* according to Livy. Sauvages comprehends under the name of *physconia,* that kind of intumescence occasioned by the preternatural increase of the solid parts of the lower belly including fat.

spect to the diversity of the different organs affected, the functions of which are variously disturbed.

Thus there are scrophulous persons who have steatomatous congestions in the internal parts, without having the glands swelled; in the same way, rachitis, which is the effect of a scrophulous habit, particularly of an hereditary taint, may produce a development more or less irregular of the body, or of some of its parts, or even a deficiency of nutrition; in such a manner that certain parts acquire an increase of size, and others lose it. This necessarily occasions diseases, which are propagated in families as the scrophulous taint is visibly transmitted when it is well characterized.

The brain in maniacs, epileptic, and apopletic persons, from their infancy, (whether the craniums of subjects that have died of these diseases, have more or less deformity, as is very common, or appear in their natural state) is always more or less hardened by steatomatous substances, and particularly the *medulla oblongata,* and the adjoining parts of the brain; as is the case in scrophula. This fact is proved by anatomical observation.

Out of the many cases of this description, with which I am acquainted, I shall only mention that of a young man who died of epilepsy, and whose mother was scrophulous, as was manifested in the glands of the neck, and who was also subject to epilepsy herself. The son died of an apoplexy after an epileptic fit, as is generally the case; and M. Markham, my assistant, opened the body. He found in the medulla oblongata, and in the productions of the brain and of the cerebellum adjoining to it, an almost cartilaginous induration; there was no apparent malconformation in the bones of the cranium.

Anatomists have frequently observed similar indurations in the brain, and sometimes also in other organs of the chest and abdomen, with swellings in the lymphatic glands, in patients who had been maniacal, or who had died of apoplexy, and whose parents expired under the same disease, without any malconformation of the cranium.

The same appearances have been found in subjects when the mind was partially and at times alienated; in these there is sometimes an apparent malconformation of the cranium, or some symptoms of a scrophulous taint, or perhaps none of these morbid affections were accompanied by external signs; but are the indurations of the brain of the same nature? It is impossible to attribute any other to them.

( To be continued. )

